# Supplementary material for: Idiopathic intracranial hypertension in patients with anemia: A retrospective observational study
Source: PLoS One. 2020 Jul 31;15(7):e0236828. doi: 10.1371/journal.pone.0236828 (PMC7394431; doi:10.1371/journal.pone.0236828)
Supplement: Attachment — Submitted filename: Response to Reviewers.docx [file pone.0236828.s001.docx]

Dear editors and reviewers,

Thanks very much for your valuable comments. For more than a month, we have carefully collected the clinical data related to the article again. Our revisions and responses to these comments are as follows:

The revised part is highlighted in blue in the manuscript.

**Journal requirements:**

When submitting your revision, we need you to address these additional requirements:

1. Please ensure that your manuscript meets PLOS ONE's style requirements, including those for file naming.

*Reply*: *My manuscript meets PLOS ONE's style requirements.*

2. In the ethics statement in the manuscript and in the online submission form, please provide additional information about the patient records used in your retrospective study, including: a) the date range (month and year) during which patients' medical records were accessed; and b) the source of the medical records analyzed in this work (e.g. hospital, institution or medical center name).

*Reply: The information have been provided in “Cohort” part of the manuscript.*

**Reviewers’ Comments**

**Reviewer #1:**
1. In general, this report provides a satisfactory picture that there is a trend that patients with anemia tend to do better. Unfortunately, the data collected is fairly minimalistic. All improvements are lumped into a simple yes or no category, and no further follow-up information is provided on the patients and their outcomes, despite having several criteria within that category as stated in the report. There is no follow-up Hb level showing that anemia was treated or not or what they were treated with, that a follow-up opening pressure was performed, or documentation of how many have papilledema and how many have shown improvement. The long-term follow-up characterization is also quite minimal and amounts to a simple “yes or no” data field and no granularity to clinical outcome data.

*Reply: The information of BCVA, headache impact, papilledema grade, automated perimetry, CSF open pressure (OP) , Hb level, treatments and improvement has been added to the “Methods” and the “Results” parts in detail.*

2. Minor issues also include incorrectly used terms (referring to an opening pressure as ICP, eg). Clinical outcomes has been converted into a subjective criteria without evidence of the objective criteria, except for visual outcomes.

*Reply: The incorrectly used terms have been revised. Accurate and objective data of clinical outcomes has been added to the “Results” part.*

**Reviewer #1:**

**Major issues:**
1. Title: The author´s annotate a tittle suggesting a retrospective cross-sectional study and later define a longitudinal cohort study. A proper analysis and definition of the type of study design is a must.

*Reply: The tittle has been revised.*

2. Materials and methods: the means of getting to the information of the patients to the database should be provided in order to validate the author´s source of research: review of data charts, radiologic tests, laboratory records, direct or indirect contact with patients, surveys, etc.

*Reply: The means of getting to the information of the participants have been provided in the “Materials and methods” part.*

3. Criteria: This paragraph could be improved greatly.
   - Diagnostic criteria should be described in the article. Even though we assume that criteria for diagnosis is homogeneous internationally, some countries could variate their way to diagnose diseases according to their data and experience.

*Reply: The Dandy’s criteria has been described in detail.*

- The course of disease was defined as acute, sub-acute and chronic. It is important to describe why and how you defined the time frame for each category, either if you chose them based on prior studies or based on your own experience. The definition for acute course needs to be added.

*Reply: The course definition was based on our own clinical experience. The authors feel that such a definition is not suitable for use in international communication, so the definition has been deleted from the text.*

- Even though it is also assumed, annotating that all patients had a complete and normal neurological exam (apart from CN VI palsy if any) is needed to ensure diagnostic criteria was met.

*Reply: A normal neurological exam has been emphasized in the text.*

- Campimetry is also part of the diagnostic criteria of IIH and a significant number of the patients did not have a campimetric study. Explanation of the missing information should be addressed, as well as the repercussions in diagnosis, treatment and response to treatment, as well as inherent limitations to the conclusions study.

*Reply: The data of campimetry, explanation of the missing information and inherent limitations have been supplemented in the article.*

4. Treatment and prognosis:
- This section analyzed the treatments given. A clear approach step by step according to response should be described. Whether the patients were recommended to lose weight initially, followed by or accompanied by farmacologic treatment and / or surgical measures (depending of the severity, duration or accompanying features of the headache). Current guidelines indicate weight control, acetazolamide 500 BID up to 2-4 g qd, furosemide 20-40 mg qd as adjunctive medication and surgical treatments. If the guidelines were not followed, an explanation of the reason as well as the aftermath in prognosis (response) and your overall conclusions of the study is recommended.
*Reply: A treatment approach step by step has been described. Intolerable side effects induced by acetazolamide is the reason of why we did not follow the guideline. The explanation has been added in the text.*

5. Citations: the author´s citations pertinent and current. However, some of them do not coincide with the information in the text. It is very important that they support and expand the assertions of fact not addressed by the data presented.

*Reply: Citations have been rearranged.*

6. Limitations: according to the aforementioned details, this section could be greatly enriched. The repercussions and possible solutions to these limitations should also be addressed.
*Reply: Limitations has been enriched as recommendation.*

**Minor issues and other comments:**
1. Discussion: Note that abbreviations and what they stand for need to be placed before using them alone in the article. It aids in a correct understanding of the text for all our readers.

*Reply: Abbreviations have been rearranged as required*

2. Figures and tables: The use of tables and / or figures may improve greatly your paper, especially for diagnostic, inclusion and exclusion criteria. A flowchart is another possibility to strengthen the readers´ understanding and focus their attention on important facts.

*Reply: Several tables and a flowchart have been added in the manuscript.*

3. Consider modifying the term renal anemia for anemia of chronic renal disease for better and correct understanding of the term.

*Reply: “Renal anemia” has been defined in detail as recommended.*

4. Image quality could be improved in order to enhance your article.

*Reply: Images has been improved.*

5. If the power of the study was calculated prior to starting the research, a description of this process is important, since it can aid in searching for an appropriate sample size and increase the chances of getting significant results. If it was not it could be added to the limitations of the study.

*Reply: The power of the study was not calculated prior to starting the research. We has listed this point in the “Limitations”.*

Sincerely,

Zhonghua Ma

2020-06-06
