# Supplementary material for: Idiopathic intracranial hypertension in patients with anemia: A retrospective observational study
Source: PLoS One. 2020 Jul 31;15(7):e0236828. doi: 10.1371/journal.pone.0236828 (PMC7394431; doi:10.1371/journal.pone.0236828)
Supplement: Attachment — Submitted filename: Response to Reviewers.docx [file pone.0236828.s002.docx]

Dear editors and reviewers,

Thanks very much for your valuable comments. Our revisions and responses to these comments are as follows:

**Journal requirements:**

“English quality should be improved in order to ease publication process, therefore, we suggest professional English editing services.”

*Reply: English quality of this manuscript has been improved.*

**Reviewers’ Comments**

“Some minor orthographic and syntactic corrections should be done prior to publication. Usually, a native English speaking aid is recommended.”

*Reply: English quality of this manuscript has been improved.*

Sincerely,

Zhonghua Ma

2020-07-06
